# Influence of hepatitis C virus on serum glutathione-S-transferase

**DOI:** 10.1186/1471-2334-14-S7-P39

**Published:** 2014-10-15

**Authors:** Mircea Niculae Penescu, Corina-Daniela Ene (Nicolae), Emanoil Ceauşu, Ilinca Nicolae

**Affiliations:** 1 Carol Davila Clinical Hospital of Nephrology, Bucharest, Romania; 2 Clinical Hospital of Infectious and Tropical Diseases “Dr. Victor Babeş”, Bucharest, Romania

## Background

Glutathione-S-transferases (GST, E.C.2.5.1.18) are a family of enzymes that catalyze the conjugation of some harmful electrophilic compounds with glutathione, and thus, they are transformed in nontoxic lipophilic substances. In this study we aimed to evaluate the effect of hepatitis C virus on serum level of GSTpi in patients with active systemic lupus erythematous (SLE).

## Methods

Serum level of GSTpi was quantified by immunoenzymatic method in 42 patients with active systemic lupus erythematous (based on SLEDAI score), without any treatment divided in two groups: Group A – 30 cases with active SLE (SLEDAI=11.2±3.2), with chronic C hepatitis; Group B – 12 cases with active SLE (SLEDAI=12.1±4.5) without hepatitis C. The results were compared to those obtained in the control group, which included 42 healthy subjects.

## Results

We determined increased levels of GSTpi in patients with active SLE without C hepatitis, when compared with the control group (236±82 versus 211±68, p>0.05). In patients from group B, the level of GSTpi was statistically significant higher than in patients from group A (286±36 versus 236±82, p<0.05), respectively in control group (286±36 versus 217±39, p<0.05).

## Conclusion

The increased levels of GSTpi in patients with SLE and chronic C hepatitis sustain the hypothesis that the release of this intracellular enzyme might be influenced by hepatitis C virus infection. This information might be useful for a better understanding of hepatitis C pathogenesis.

